# A comparison of international modelling methods to evaluate health economics of colorectal cancer screening: a systematic review protocol

**DOI:** 10.1186/s13643-023-02173-w

**Published:** 2023-01-27

**Authors:** Olivia Adair, Ethna McFerran, Tracy Owen, Christine McKee, Felicity Lamrock, Mark Lawler

**Affiliations:** 1 Mathematical Sciences Research Centre, Queen’s University Belfast, Belfast, Northern Ireland; 2 Patrick G Johnston Centre for Cancer Research, Queen’s University Belfast, Belfast, Northern Ireland; 3 Public Health Agency, 12-22 Linenhall Street, Belfast, BT2 8BS UK

**Keywords:** Colorectal cancer, Screening, Cost-effectiveness analysis, Cost-utility, Cost–benefit, Quality-adjusted life years, Life years gained, Incremental cost-effectiveness ratio, Economic evaluation, Health economics

## Abstract

**Background:**

Colorectal cancer (CRC) is becoming an increasing health problem worldwide. However, with the help of screening, early diagnosis can reduce incidence and mortality rates. To elevate the economic burden that CRC can cause, cost-effectiveness analysis (CEA) can assist healthcare systems to make screening programmes more cost-effective and prolong survival for early-stage CRC patients. This review aims to identify different CEA modelling methods used internationally to evaluate health economics of CRC screening.

**Methods:**

This review will systematically search electronic databases which include MEDLINE, EMBASE, Web of Science and Scopus. The Preferred Reporting Items for Systematic Reviews and Meta-Analysis (PRISMA) guidance recommendations will design the review, and the Consolidated Health Economic Evaluation Reporting Standards (CHEERS) statement will be used to extract relevant data from studies retrieved. Two reviewers will screen through the evidence using the PICOS (Participant, Intervention, Comparators, Outcomes, Study Design) framework, with a third reviewer to settle any disagreements. Once data extraction and quality assessment are complete, the results will be presented qualitatively and tabulated using the CHEERS checklist.

**Discussion:**

The results obtained from the systematic review will highlight how different CRC screening programmes around the world utilise and incorporate health economic modelling methods to be more cost-effective. This information can help modellers develop CEA models which can be adapted to suit the specific screening programmes that they are evaluating.

**Systematic review registration:**

PROSPERO CRD42022296113

**Supplementary Information:**

The online version contains supplementary material available at 10.1186/s13643-023-02173-w.

## Background

Colorectal cancer (CRC) is the second most common cancer in females and the third most common cancer in males throughout the world [1]. However, morbidity and early death of CRC can be prevented through early diagnosis, which can be enhanced via screening. Although extremely beneficial to the public, screening and the subsequent clinical pathways leading from this approach can be costly. In 2015, it was found that across Europe, the economic burden of CRC was €19.1 billion [2]. To maximise the survival and long-term effects on the public’s health and potentially reallocate the costs attributable to CRC towards other parts of the economy, a cost-effective approach is needed.

CRC most commonly develops from polyps in the colon and/or rectum [3]. These polyps can grow within the glandular tissue which is located in the intestinal lining. Most polyps are benign; however, with increased numbers and size [4], polyps are known to develop into precancerous diseases via the adenocarcinoma sequence where genetic changes can arise [5]. Whilst colonoscopy is considered to be the gold standard tool of screening with a high sensitivity and specificity [6], non-invasive stool tests are commonly used in screening across Europe [7], with faecal immunochemical testing (FIT) most commonly used to detect any haemoglobin within the stool. If this test is positive, a colonoscopy is performed to examine the bowel. If polyps are identified during a colonoscopy procedure, they can be removed before developing into cancer. The primary aim of CRC screening programmes is to diagnose cancer early. Thus, to reduce the occurrence of later stages of this disease, screening programmes have been introduced to help tackle this public health problem [1].

CRC screening is subject to the World Health Organization (WHO) principles of screening, as first discussed by Wilson and Jungner [8], whereby understanding the interplay of the harms and benefits of the ability to test for the pre-clinical disease is a necessary step in health planning. Decision-makers need to consider the most cost-effective approach for their service in order to best understand whether a non-invasive diagnostic test, such as FIT, at a particular threshold, is cost-effective within a limited capacity [9]. Crucially, these screening principles have led to the application of cost-effectiveness analyses to evaluate screening and support decision-makers in their planning and delivery of services. However, without a full understanding of the CEA modelling methods and how their use influences the delivery of screening programmes and future decision-making, unintentional bias may result from incorrectly using a modelling method that does not best capture the decision problem [10]. Improving the understanding of the modelling approaches taken to evaluate screening programmes should create greater transparency and context for researchers, decision-makers and ultimately patients within screening programmes.

This review protocol will create a comprehensive search of cost-effectiveness analysis modelling methods for CRC screening programmes used internationally. This information will be used to examine and compare the key attributes of cost-effectiveness models. The findings will support future efforts to build more efficient models, grounded in real-world data for the evaluation of screening programmes worldwide.

## Methods

The protocol of this systematic review adheres to the Preferred Reporting Items for Systematic Reviews and Meta-Analysis Protocols (PRISMA-P 2015) [11] guidelines (see Additional file 1). The protocol is also registered in the PROSPERO database, the International prospective register of systematic reviews (CRD42022296113).

The PRISMA [12] guidance recommendations have been used to design the systematic review. The systematic review will apply the Consolidated Health Economic Evaluation Reporting Standards (CHEERS) [13] statement to extract relevant data from studies retrieved.

### Decision problem review question

The objective of this review is to identify the modelling methods and assumptions used for colorectal cancer screening globally and to derive cost-effective analysis/cost-utility analysis/cost–benefit analysis outcomes. The following specific questions will be addressed in the review.

1. What type of model (for example, Markov, semi-Markov, discrete event simulation) and model structure is used in a particular CRC screening programme?
2. What are the model assumptions for each model structure, e.g. time horizons, LYGs, QALYs, costs and other parameters?
3. What is the associated background mortality with the population used for the building of this model? Are other model parameters included, such as efficiency parameters?

### Defining inclusion criteria

A PICOS (Participants, Interventions, Comparator, Outcomes and Study Design) framework was used for the inclusion and exclusion criteria. The study design was specified since randomised controlled trials and cohort studies were deemed unlikely to provide suitable reports on the cost-effectiveness modelling of screening strategies.

- Participants—males and females at average risk of CRC, or individuals eligible to receive a CRC screening invitation for a given screening programme
- Interventions—interventions for screening for CRC, for example, faecal immunochemical test (FIT), faecal occult blood test (FOBT), flexible sigmoidoscopy, colonoscopy, computed tomography colonography (CTC), double-contrast barium enema or any other screening methods, which are not included in this list
- Comparators—we expect the main comparator in most papers to be no screening compared to various screening strategies, for example, varying age ranges of the screening population and thresholds associated with quantitative FIT testing and FOBT testing
- Outcomes—incremental cost-effectiveness ratio (ICER), net benefit, life years gained (LYGs), quality-adjusted life years (QALYs), costs, colonoscopy utilisation or any other unit of health gain and screening harm
- Study designs—all economic evaluation academic papers which are published and discuss an economic evaluation model are eligible for inclusion in the review

### Defining exclusion criteria

- Participants—only human participants are to be included in the papers and not animals
- Interventions—interventions used outside of the interventions listed above
- Study designs—to avoid potentially high bias, case reports and case series are excluded from this review. Randomised controlled trials and cohort studies are needed for the quality evaluation of models but are not directly necessary for this review
- Time frame—academic papers published between January 2011 and December 2021

### Identifying research evidence

The search strategy was optimised following advice from the Specialist Medical Librarian at the host institution, who suggested using several databases to meet the needs of the review. The databases used for the planned strategy were MEDLINE, EMBASE, Web Of Science and Scopus. Searches were conducted in December 2021 using the.

MESH search terms (see Additional file 2) and updated in January 2022 for the following:

1. Colorectal Neoplasms OR colorectal cancer OR bowel cancer OR colorectal
2. screen* OR Mass Screening
3. Cost–Benefit Analysis OR cost-effective* OR cost-utility OR quality-adjusted life-years OR life-year* gain* OR economic evaluat* OR health technology assessment OR incremental cost-effective* ratio* OR ICER* OR cost analys* OR qaly* OR lyg*

Searches 1, 2 and 3 are then combined by AND. Only English papers were included in this review since there was no funding to translate non-English language papers. The studies which fulfil this criterion were then screened for inclusion by the PICOS criteria.

### Study selection

There will be three stages in the study selection process.

1. All results found from the initial search will be downloaded into the COVIDENCE systematic review manager [14] to facilitate the review management, including the removal of duplicated articles before starting the title and abstract review.
2. The titles and abstracts of the papers will be screened by two reviewers following the PICOS criteria.
3. The full text of the eligible papers will be reviewed to further confirm their eligibility.

If the two reviewers disagree on papers, a third reviewer will be introduced and a consensus of the three reviewers will be made. Also, in the case that multiple papers report the same model/method, the most recent paper with the overall model structure will be included, thus resulting in other papers with this model being discarded. The papers excluded will be deemed ineligible according to the PICOS criteria.

A sample of papers will be used to pilot the selection process using the inclusion criteria, to determine whether papers are being selected appropriately. If any noticeable or fundamental changes need to be addressed to the inclusion criteria, they can be iteratively improved by the reviewers to support the objectives.

### Data extraction

The extracted data will use the CHEERS checklist as a benchmark to guide the collation of data, for example, extracting the study population, perspective, time horizon and discount rate from the papers. Data extraction will also focus on the model design, parameter selection and modelling methods to examine modelling practices and understand the sources of variation in methods, the likely impact on study quality and therefore its appropriateness for future application. Unfortunately, the CHEERS checklist does not include any items about potential bias within the studies. This is an important potential source of error in modelling and parameter assumptions can introduce unintended biases when reporting the results of the model [15]. This will be a limitation of the paper and will be addressed within the discussion of the review.

### Quality assessment

This protocol aims to collate reported methods rather than examine their quality. Therefore, instead of using the Drummond checklist [16] which examines the methodological quality, the CHEERS checklist will be used for quality reporting of economic evaluations [17] as recommended by the EQUATOR Network [18]. Papers eligible will undergo quality assessment, where detailed information on study characteristics will be collected, based on the CHEERS guideline checklist.

### Data synthesis

A PRISMA flow chart showing the number of papers remaining at each stage will be presented to document the study selection process.

As the purpose of this review is to examine the modelling methods used to evaluate the health economics of CRC screening, the results will be presented qualitatively. The studies included in the final review will be tabulated by the CHEERS checklist. Some key features will include the type of study, outcome measures and interventions. Thus, methodological study quality will be addressed, for example, the economic model choice, effectiveness measure and cost measures. Examination of the results will also collate any sensitivity analyses performed in the studies to help identify if there are any changes to the study conclusions due to the susceptibility of cost-effectiveness to key model parameters.

### Dissemination

Results found from this systematic review will be published in a peer-reviewed journal and disseminated at international conferences and institutional academic workshops.

## Discussion

Earlier diagnosis of colorectal cancer as a result of screening can result in improved treatment and survival outcomes. To achieve this goal, healthcare providers can use health economic modelling methods to develop a more cost-effective approach to tackling CRC screening and tailor the methods specifically to other countries. This review will collate the modelling methods and assumptions used in CRC screening programmes internationally and thus can be a guide for modellers, for future design and to illustrate improvements, standardisation and quality evaluation of the models used within screening programmes to date.

The papers will be screened using the PICOS framework in this systematic review protocol, whilst the data extraction and reporting assessment will follow the CHEERS checklist [13]. One limitation of this review, which may result in publication bias, is that only English language papers will be used since there was no funding for non-English papers to be translated. During the data synthesis process, the results will be qualitatively reported and tabulated in line with the CHEERS checklist. Therefore, the findings should give modellers and policymakers better insight into the modelling methods available within colorectal cancer screening, and how best to tailor such models for a defined population.

## Supplementary Information

**Additional file 1.** PRISMA-P Document 2015. PRISMA-P document is used to make the peer review process efficient and strengthen the quality of reporting.

**Additional file 2.** MESH Search Terms. This document provides the MESH search terms that were included specifically in the MEDLINE database and altered to fit the other databases.

## Data Availability

Not applicable.

## Abbreviations

CRC: Colorectal cancer
CEA: Cost-effectiveness analysis
PRISMA: Preferred Reporting Items for Systematic Reviews and Meta-Analysis
CHEERS: Consolidated Health Economic Evaluation Reporting Standards
PICOS: Participants, Interventions, Comparators, Outcomes, Study Designs
WHO: World Health Organization
LYGs: Life years gained
QALYs: Quality-adjusted life years
FIT: Faecal immunochemical test
FOBT: Faecal occult blood test
CTC: Computed tomography colonography
ICER: Incremental cost-effective ratio
