# Supplementary material for: A comparison of international modelling methods to evaluate health economics of colorectal cancer screening: a systematic review protocol
Source: Syst Rev. 2023 Jan 27;12:14. doi: 10.1186/s13643-023-02173-w (PMC9883863; doi:10.1186/s13643-023-02173-w)
Supplement: Supplementary file 2 — Additional file 2. MESH Search Terms. This document provides the MESH search terms that were included specifically in the MEDLINE database and altered to fit the other databases. [file 13643_2023_2173_MOESM2_ESM.pdf]

Mesh Search terms shown in the databases

The following keywords were taken from the MEDLINE database. However, they were also used in the EMBASE, Web of Science and Scopus databases.

1. exp Colorectal Neoplasms/
2. colorectal cancer.mp
3. bowel cancer.mp
4. colorectal.mp
5. screen*.mp
6. Mass Screening/
7. Cost-Benefit Analysis/
8. cost effective*.mp
9. cost utility.mp
10. quality-adjusted life years/
11. life year* gain*.mp
12. economic evaluat*.mp
13. health technology assessment.mp
14. incremental cost effective* ratio*.mp
15. ICER*.mp
16. cost analys*.mp
17. qaly*.mp
18. lyg*.mp
19. 1 or 2 or 3 or 4
20. 5 or 6
21. 7 or 8 or 9 or 10 or 11 or 12 or 13 or 14 or 15 or 16 or 17 or 18
22. 19 and 20 and 21
23. limit 22 to yr="2011 -Current"
24. limit 23 to english language
25. limit 24 to humans
26. limit 25 to (classical article or "corrected and republished article" or introductory journal article or journal article or preprint or "review" or "scientific integrity review")
